# A rare case of a double phytobezoar causing gastric and jejunum obstruction in an adult man: a case report

**DOI:** 10.1186/s13256-016-1137-7

**Published:** 2016-12-15

**Authors:** S. Occhionorelli, M. Zese, S. Targa, L. Cappellari, R. Stano, G. Vasquez

**Affiliations:** 1 Department of Morphology, Surgery and Experimental Medicine, University of Ferrara and Sant’ Anna Universitary Hospital of Ferrara, Ferrara, Italy; 2 Department of Surgery, Emergency Surgery Service, Sant’Anna University Hospital, Ferrara, Italy

**Keywords:** Bezoars, Phytobezoars, Small bowel obstruction, Gastrostomy, Enterotomy

## Abstract

**Background:**

Bezoars are an uncommon cause of mechanical intestinal occlusion. There are four different kinds of bezoars: phytobezoars, made of vegetables and fibers; trichobezoars, resulting from the ingestion of hair and frequently an expression of psychiatric disorders; lactobezoars, which are formed of milk curd; and pharmacobezoars, caused by drugs and medications. Symptoms are classically indistinguishable from one another and from more common causes of intestinal occlusion, so it can be difficult to establish a correct diagnosis in order to apply the correct treatment. We present a rare case of two different phytobezoars causing intestinal occlusion (gastric and jejunal). We also describe the correct techniques for making a correct and fast diagnosis of occlusion caused by phytobezoars, and the possible conservative and operative treatments.

**Case presentation:**

We present the case of a double phytobezoar that was surgically treated with a double enterotomy. Our patient was a 68-year-old Caucasian man with a medical history of hypertension, a previous open appendectomy, and open repair of a perforated gastric ulcer. He was admitted with a 5-day history of abdominal pain located in his upper quadrants along with vomiting. After a preoperative examination, he was taken to the operating room. He was discharged in a good clinical condition 11 days after surgical intervention. A physical examination at 6 months demonstrated our patient was in good health.

**Conclusions:**

Diagnosing bezoars is difficult because of their rarity. However, they must be taken into consideration in a differential diagnosis because their treatment is not always surgical. In fact, it may be conservative in many cases and a correct diagnosis will guide towards the correct therapy.

## Background

Small bowel obstruction (SBO) is a common surgical presentation, with different possible etiologies that include postoperative adhesions (60–80%), volvulus, intussusceptions, hernias, and tumors. In all these cases symptoms are similar and include abdominal pain, nausea and vomiting, inability to pass gas or bowel movements, and sometimes fever [1], so a correct differential diagnosis is important. Bezoars represent an infrequent cause of mechanical SBO and can occur in 0.4–4% of total cases, according to different authors [2–4]. Rarely, they can form and stop in the stomach and can cause ulcerative lesions, bleeding, or, very infrequently, obstruction [5]. Bezoars are classically defined as an intraluminal solid foreign body, made of different indigestible materials [6–9]. There are four different types, named after the material from which they are composed: trichobezoars, resulting from the ingestion of hair; phytobezoars, made of vegetables and indigestible fruit fibers; lactobezoars, which are formed from milk curd; and pharmacobezoars, caused by drugs and medications [6, 10].

Trichobezoars occur especially in psychiatric disorders, such as trichotillomania and trichophagia. They are more frequent in young women and frequently located in the stomach, with a possible extension up to the ileocecal junction [5, 6, 11, 12]. Lactobezoars affect milk-fed infants; they are associated with multifactorial events but the incidence has been declining in the last few years [5]. Pharmacobezoars are generally caused by sodium polystyrene sulfonate (Kayexalate), cholestyramine, and antacid drugs [6, 13]. Phytobezoars are the most common type, usually impacting the narrowest portion of the small bowel [14]. Predisposing factors include prior gastric surgery, chronic gastritis, Crohn’s disease, gastrointestinal carcinoma, dehydration, hypothyroidism, advanced age, diabetes, neuropathy, or myotonic dystrophy [15, 16]. Furthermore, an excessive ingestion of cellulose-containing foods, insufficient mastication, poor dental hygiene, drugs, or other myotonic diseases can lead to their formation [16].

Symptoms such as abdominal pain and distension, nausea and vomiting, dysphagia, weight loss, fever, and constipation [1, 6, 17, 18] are nonspecific for SBO. This unspecificity complicates the diagnosis owing to the low incidence of bezoars. In this paper, we report a rare case of an adult man with a double phytobezoar, treated with surgical laparotomy.

## Case presentation

**Fig. 1:**
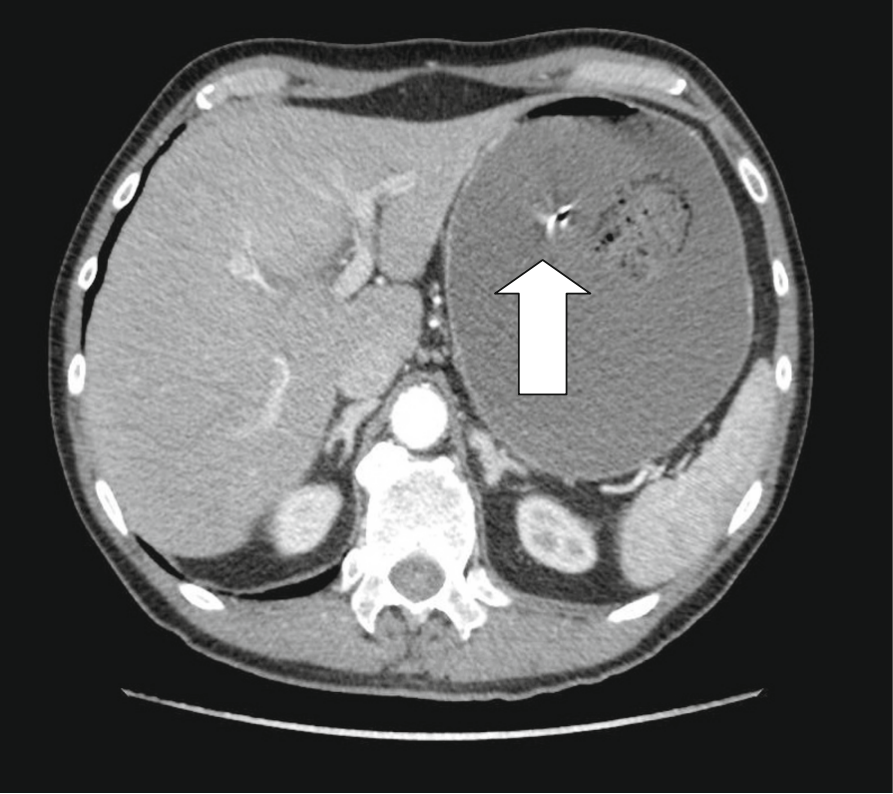
Preoperative abdominal computed tomography scan. The *arrow* indicates the intraoperative view of gastric phytobezoar while removing

A 68-year-old Caucasian man was admitted to our emergency surgery with a 5-day history of abdominal pain located to his upper quadrants along with vomiting. Our patient’s history included hypertension, a previous open appendectomy, and open repair of a perforated gastric ulcer. He described the pain as a worsening continuous ache lasting several hours, relieved by taking prokinetic agents and anti-emetics. A physical examination revealed a slightly distended abdomen. Deep palpation caused pain in his left quadrants but there were no signs of peritoneal or gallbladder inflammation; both Murphy and Blumberg signs were negative. Auscultation revealed active peristalsis. His laboratory test results were significant for neutrophilic leukocytosis (21,900/mm^3^; normal, 19,900/mm^3^) with signs of hemoconcentration (hemoglobin 15.5 g/dL; hematocrit 45%). His liver and renal function were normal, with a creatinine concentration of 0.99 mg/dL. An abdominal X-ray revealed signs of bowel obstruction with jejunal air-fluid levels. An abdominal computed tomography (CT) scan with contrast medium showed massive distension of his stomach, duodenum, and jejunum with an abrupt stop at that point: the remaining loops of his intestine appeared to have collapsed. Particularly, his stomach and the lower third of his esophagus were distended by abundant fluid material and there was no evidence of free air in his abdomen and no peritoneal fluid (Fig. 1).

**Fig. 2:**
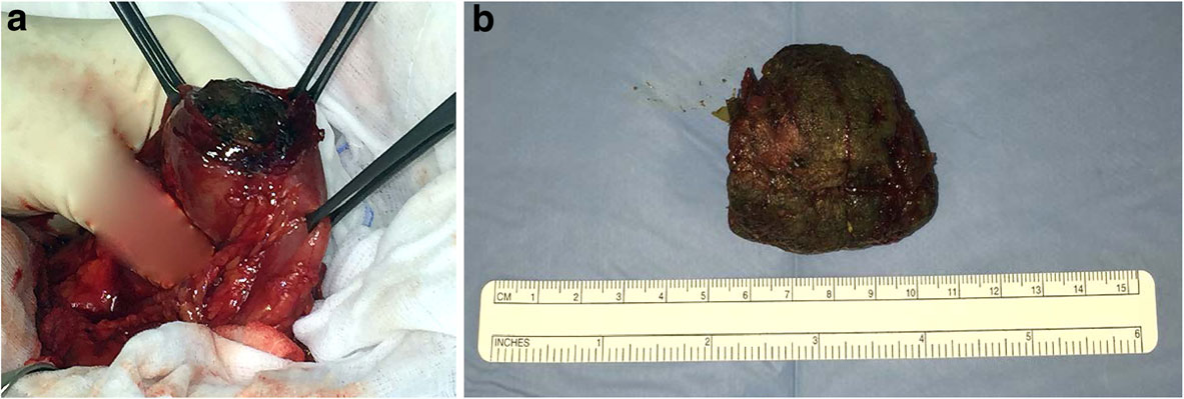
**a** Intraoperative view of gastric phytobezoar. **b** Gastric phytobezoar

**Fig. 3:**
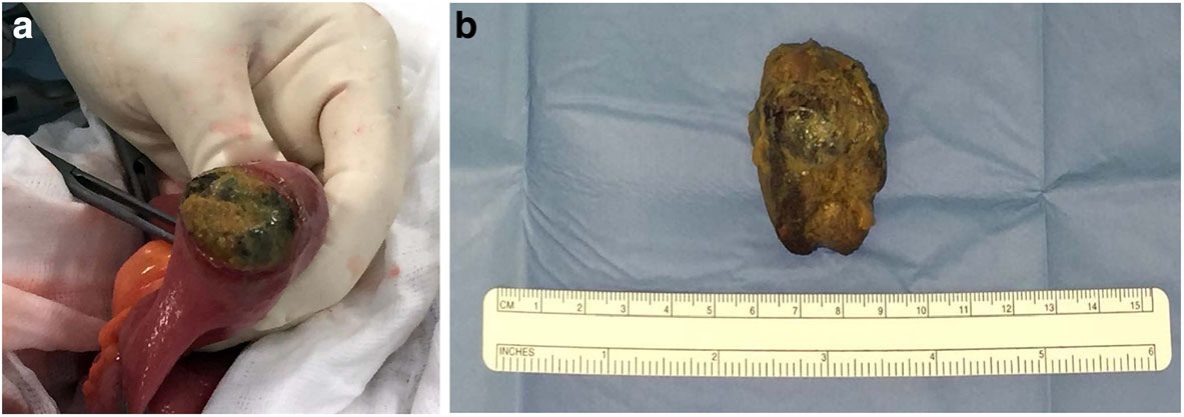
**a** Intraoperative view of jejunum phytobezoar. **b** Jejunum phytobezoar

Taking into account his clinical symptoms, laboratory test results, and the instrumental evaluation, urgent explorative laparotomy was performed. This revealed abnormal distension of his stomach and jejunum, with two foreign bodies of diameter 4 cm, one in the gastric body (Fig. 1) and one in the distal part of his jejunum. After gastrostomy and jejunostomy, the foreign bodies were extracted and the incisions were sutured (Figs. 2 and 3). Time to bowel movement and time to bowel activity were particularly slow in the postoperative days. An abdominal X-ray was performed on the sixth postoperative day after an iodinated contrast medium was administered through a nasogastric probe. This demonstrated complete opacification of his colon. Our patient was discharged 11 days after surgery in a good clinical condition. A pathologic examination of the intraluminal lesion revealed the presence of a double phytobezoar made of different types of fibers. Our patient attended a follow-up visit after 6 months, and a physical examination evidenced that he was in a good clinical condition.

## Discussion

Phytobezoars are an uncommon etiology of SBO. They can be suspected in patients with co-morbidities or psychiatric diseases but diagnosis in healthy patients can be difficult, in particular if diagnosis is based solely on a physical examination and anamnesis [19]. In the presented case we found two phytobezoars located in different regions of our patient’s bowel. The first occluded his stomach while the second occluded his jejunum, causing a double air-fluid level with significant gastric distension. Results from blood tests, in particular the presence of leukocytosis, were completely nonspecific and did not help us in our diagnosis. According to the literature, phytobezoars can be diagnosed using abdominal radiography, which can highlight air-fluid levels associated with mechanical obstruction. However, many authors agree that it is nonspecific [6, 20]. Some authors assert that bezoars create hyperechoic acoustic shading in ultrasonography and that this tool can recognize them in 88–93% of ileal localizations [6, 21, 22]. Its use is controversial because it is operator dependent, patient dependent, and has low sensitivity [23]. A barium enema or endoscopy can also be used, although their diagnostic accuracy is limited [1]. CT with contrast enhancement, which has a sensitivity of 90% and a specificity of 57% in recognizing bezoars [6, 22, 24, 25], is now the gold standard in the diagnosis of bezoars and SBO. It permits the differential diagnosis of other bowel masses [19] and identifies signs such as ascites, wall bowel thickening, proximal lumen dilatation, and intestinal infarction [1]. CT can aid in choosing a conservative or a surgical/endoscopic treatment strategy; in most cases of bezoars and SBO its leads to a targeted surgical therapy. In our case, our patient’s history and CT images initially drove us to the diagnosis of tumoral gastric occlusion – bezoars were not at this time considered. For this reason, we decided on a median laparotomy, which allowed us to determine the real cause of his SBO.

The treatment of bezoars can be conservative, especially in the case of phytobezoars. Mechanical disintegration can be tried, using mechanical lithotripsy, a Dormia basket, or an electrosurgical knife [6]. Chemical dissolution is another option, with Coca-Cola® lavages or hydrolytic solutions [5]. Some authors have reported good results in the application of both methods [26]. Other operative treatments depend on the size, consistency, and location of the bezoar. Small bezoars can be removed with endoscopic treatment [27]. With larger bezoars, which can cause occlusion or bleeding, surgery is usually necessary. In these cases, laparoscopy or laparotomy is mandatory [5, 12]. In our case, the nonspecific etiology led us to perform a laparotomy. There are different options for SBO treatment, including manual fragmentation and pushing the bezoar through the bowel as far as the caecum [2, 10], removal per anum, or enterotomy. Bowel resection and anastomosis is required when occlusion is complicated by transmural ischemia [12], but this was not the case in our patient, thus we decided to perform two enterotomies.

## Conclusions

Bezoars must be taken into consideration in cases of SBO even though they are infrequent. Their presentation classically is indistinguishable from other causes of occlusion. In cases in which the nature of the occlusion is known, the treatment could be nonsurgical. The case presented in this article is interesting because bezoars were not suspected. However, in our particular case, the choice of treatment would have been the same, even if starting with the correct diagnosis. It must be noted that surgery may not always be the best course of treatment. Therefore, it is important to make an accurate diagnosis in order to adopt the most effective strategy and consider every possible solution.

## Abbreviations

CT: Computed tomography
SBO: Small bowel obstruction
